# Where Did They Come from—Multi-Drug Resistant Pathogenic *Escherichia coli* in a Cemetery Environment?

**DOI:** 10.3390/antibiotics7030073

**Published:** 2018-08-14

**Authors:** Akebe Luther King Abia, Eunice Ubomba-Jaswa, Chantelle Schmidt, Matthys Alois Dippenaar

**Affiliations:** 1 Antimicrobial Research Unit, College of Health Sciences, University of KwaZulu-Natal, Private Bag X54001, Durban 4000, South Africa; lutherkinga@yahoo.fr; 2 Water Research Commission, Private Bag X03 Gezina, Pretoria 0031, South Africa; 3 Department of Biotechnology, University of Johannesburg, Doornfontein, Johannesburg 2094, South Africa; 4 Engineering Geology and Hydrology, Department of Geology, University of Pretoria, Pretoria 0084, South Africa; schmidtchantelle@gmail.com

**Keywords:** cemetery, pathogenic *E. coli*, multi-drug resistance, antibiotic resistance, environmental reservoirs, public health

## Abstract

Human burial in cemeteries facilitates the decomposition of corpses without posing a public health danger. However, the role of cemeteries as potential environmental reservoirs of drug-resistant pathogens has not been studied. Thus, we investigated cemeteries as potential environmental reservoirs of multi-drug resistant (MDR) pathogenic *Escherichia coli*. *E. coli* isolates were obtained from water samples (collected from surface water bodies and boreholes in three cemeteries) after isolation using the Colilert^®^ 18 system. Pathogenic potentials of the isolates were investigated using real-time polymerase chain reactions targeting seven virulence genes (VGs) pertaining to six *E. coli* pathotypes. The resistance of isolates to eight antibiotics was tested using the Kirby–Bauer disc diffusion method. The mean *E. coli* concentrations varied from <1 most probable number (MPN)/100 mL to 2419.6 MPN/100 mL with 48% of 100 isolates being positive for at least one of the VGs tested. Furthermore, 87% of the isolates were resistant to at least one of the antibiotics tested, while 72% of the isolates displayed multi-drug resistance. Half of the MDR isolates harboured a VG. These results suggest that cemeteries are potential reservoirs of MDR pathogenic *E. coli*, originating from surrounding informal settlements, which could contaminate groundwater if the cemeteries are in areas with shallow aquifers.

## 1. Introduction

There is a growing concern regarding the global increase in antibiotic-resistant microorganisms. These antibiotic-resistant organisms have a major impact on public health as they have caused an increase in therapeutic failures [1], hospitalisation [2] and death rates [3]. There has also been a remarkable re-emergence of neglected diseases, such as extremely drug-resistant tuberculosis [4]. Although some bacteria are resistant to a single antibiotic, multi-drug-resistant organisms have developed simultaneous resistance to several antibiotics [5,6]. To face this challenge, several combined therapies have been developed with different spectrums of activity [7,8]. Unfortunately, resistance to many combined therapy formulations has also been reported [9,10].

Although antibiotic resistance can be intrinsic, a strong association between human activities and the occurrence of acquired antibiotic resistance has been observed in the environment. Activities, such as the discharge of untreated or poorly treated sewage into receiving water bodies [11], use of sewage sludge in agriculture [12] and use of animal faeces as fertiliser [13], have been linked to the increased detection of antibiotics and antibiotic-resistant bacteria in the environment. Despite their effectiveness in removing bacteria from wastewater, wastewater treatment works, for example, are unable to completely remove antibiotics from wastewater, thus resulting in the discharge of these metabolically active compounds into the environment [14]. It has been suggested that microbial exposure to these sub-lethal doses of antibiotics leads to the development of resistance to the antibiotics in bacteria [15,16].

Another public health concern associated with environmental pollution due to human activities is the introduction of pathogens into the environment. These pathogenic organisms, which include bacteria, viruses, fungi and protozoa [17], have been proven to be involved in many human and animal diseases. One such organism that has been studied extensively is *Escherichia coli*. Although initially regarded as a harmless member of the microflora in the gut of humans and other warm-blooded animals, *E. coli* is now represented by non-pathogenic and pathogenic strains. In humans, pathogenic *E. coli* strains may cause intestinal and extraintestinal infections [18,19]. The strains that are involved in intestinal infections, which are collectively termed Diarrhoeagenic *E. coli* (DEC), have gained greater attention because of their association with numerous high-profile diarrhoeal disease outbreaks worldwide, including in developed countries. For example, Shiga toxin-producing *E. coli* O157 (STEC O157) was implicated in two disease outbreaks in multiple states in the United States of America in 2008 [20]. A similar outbreak had earlier been reported in Sweden in 2002 [21]. In South Africa, the extraintestinal uropathogenic *E. coli* was the most common isolated strain involved in urinary tract infections in private and public clinics, accounting for over 80,000 cases between 2007 and 2011 [22]. Pathogenic *E. coli* strains have also been reported to be resistant to numerous antibiotics [23]. 

One inevitable phase of every living being is death. In the human population, death is usually accompanied by diverse funeral practices and rituals depending on the community involved [24]. Although it has been reported that some communities practise building or settlement burials (usually to keep them close to their loved ones), most burials are carried out in cemeteries, which are specific areas dedicated for the purpose [25]. As such, cemeteries are regarded as historical monuments where people usually go to remember their lost loved ones [26]. The burial of human remains in cemeteries is said to facilitate the decomposition of the corpse without posing a danger to public health. However, it is believed that cemeteries could represent a health risk. In particular, this risk is greatest in grave diggers due to injuries or contamination of wounds during digging of the graves [27]. In South Africa, studies addressing graveyards [28] and the associated potential health risk are rare, which is similar to the situation in many other countries. The potential contribution of burial in cemeteries as a human activity to the presence of drug-resistant microbial pathogens in the environment has not been given full attention. Thus, the current study was conducted to investigate cemeteries as potential environmental reservoirs of multi-drug resistant pathogenic *E. coli* as these organisms could represent a potential health threat through the contamination of groundwater.

## 2. Results

### 2.1. Enumeration of Escherichia coli

The two surface water and two of the eight groundwater samples from the Maitland cemetery were positive for *E. coli* (Table 1). The mean *E. coli* concentrations from the positive Maitland cemetery samples ranged from 5.2 and 11.5 MPN/100 mL for the groundwater and 2419.6 MPN/100 mL for the surface water.

All samples collected from the Delft cemetery were negative for *E. coli*, while only the stream samples were positive for the Welmoed cemetery. Some of the sites indicated on the maps were not analysed as water was not intercepted during the digging of the boreholes.

### 2.2. Virulence Potentials of Isolates

**Figure 1:**
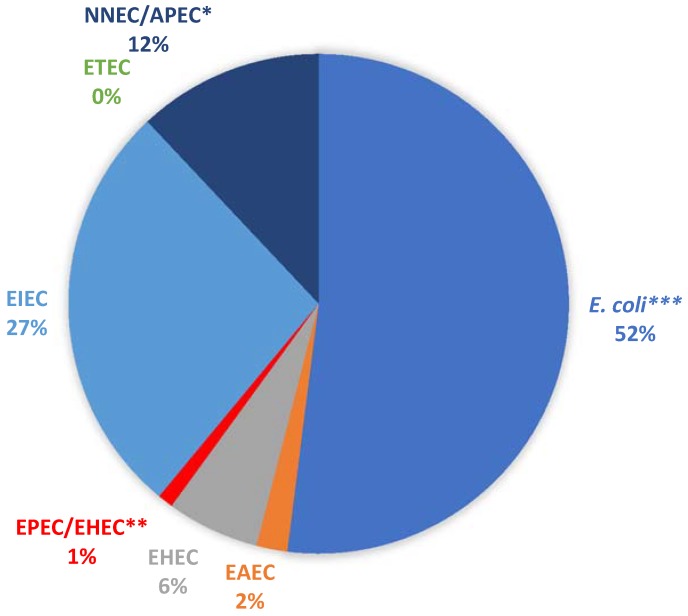
Percentage detection of the various *E. coli* pathotypes. * NNEC/APEC: positive for the *ibeA* gene, which is present in both Avian Pathogenic *E. coli* (APEC) and NMEC (Neonatal Meningitis-Associated *E. coli*). ** EPEC/EHEC (Enteropathogenic *E. coli*/Enterohaemorrhagic *E. coli*): positive for the *eaeA* gene, which is present in both pathotypes. *** *E. coli* denotes all isolates that were not positive for any of the Virulence Genes tested.

A total of 100 presumptive *E. coli* isolates (19 from borehole and 81 from surface water samples) were randomly selected from the pure culture plates and profiled for their virulence potentials. All the isolates were positive for the *mdh* gene, confirming them as *E. coli*. In total, 48 of the isolates were positive for at least one of the pathotypes examined (Figure 1).

Five isolates carrying at least one of the VGs were from the positive borehole water sample. Four of these isolates were EIEC (Enteroinvasive *E. coli*), while one was NMEC/APEC (Neonatal Meningitis-Associated *E. coli*/Avian Pathogenic *E. coli*). Most of the samples (52%) did not carry any of the genes tested. Of those carrying at least one of the genes, the EIEC group was the most abundant, while the ST gene of ETEC (Enterotoxigenic *E. coli*) was not detected in any of the pure isolates.

### 2.3. Antibiotic Resistance Profiles of Isolates

All the *E. coli* isolates were tested for resistance against eight antibiotics. We found that 87 of these were resistant to at least one of the antibiotics tested, with the highest resistance observed against Streptomycin (71%) and Trimethoprim (71%) (Table 2). Only seven of the resistant isolates were from the borehole water samples.

To determine the various resistant phenotypes and multiple-antibiotic resistance, only isolates that demonstrated complete resistance were included in the calculations. Multi-drug resistance (MDR) was considered as resistance to three or more antibiotic classes. Only one isolate (1/87) was resistant to a single antibiotic (C/T), while 72/87 were MDR (Table 3).

Most of the isolates (22/87) were resistant to four different antibiotics. The most abundant phenotypic group was the S-T-TM-CIP with 13 isolates, 4 of which were from the borehole samples. The remaining four resistant isolates from the borehole samples displayed the S-T (2) and S-T-TM-CIP-C/T (2) phenotypes. Four isolates were resistant to all eight antibiotics tested.

Forty-one of the resistant isolates (41/87; 47.13%) were positive for at least one of the VGs tested, while 36 of all the MDR (36/72; 50%) were also pathogenic. None of the resistant isolates from the borehole samples had pathogenic potentials based on the VGs tested in this study.

## 3. Discussion

Cemeteries are dedicated pieces of land used for burying human cadavers and facilitate the transformation of the dead bodies in a manner that aims to eliminate public health dangers. These places have also been regarded as places where people visit to remember their loved ones and as symbols of the historical memory of a given society [26]. Despite their importance to humanity, these preserved areas could serve as environmental reservoirs of drug-resistant pathogenic microorganisms. The current study investigated the presence of multi-drug resistant pathogenic *E. coli* in selected cemeteries in Cape Town, South Africa. The results of the study showed that pathogenic strains of *E. coli* were present in the cemeteries being studied and a substantial percentage of the pathogenic strains were also resistant to three or more of the eight antibiotics tested.

### 3.1. Enumeration of Escherichia coli

We recorded the highest *E. coli* count per sample (2419.6 MPN/100 mL) in Maitland cemetery during the study. This site also recorded the highest number of positive samples compared to the other sites and was the only site with borehole water samples that tested positive for *E. coli*. However, the microbial counts in the borehole samples were far lower compared to the surface water samples.

Maitland cemetery was the largest of the three cemeteries included in the study and was surrounded by more human habitats compared to the Delft and Welmoed cemeteries. Most of the settlements around the cemetery were informal, usually lacking basic sanitary facilities. As a result, the areas surrounding the cemetery are frequently used for defaecation and dumping of household refuse. Therefore, this could explain the fact that the surface waters in the cemetery recorded the highest number of *E. coli*. It could be possible that the bacteria originated from the runoff from the surrounding informal settlements. Informal settlements have been recognised as important contributors to environmental pollution and it has been recommended that providing these settlements with adequate sanitary facilities could help to prevent such pollution [29]. Rodents and other animals, including domestic animals, also harbour *E. coli* in their guts [30,31,32]. Given that the cemetery environment is not frequently visited by humans, these areas could serve as good habitats for these animals, which may also contribute to the pollution of the environment. However, given the elevated bacterial counts observed in the current study, it is unlikely that animals were a significant contribution to the *E. coli* counts recorded.

The number of sampling points was determined by the size of the cemetery. As such, being the largest of the three cemeteries, more sampling points were located in the Maitland cemetery. This could have increased the chances of recording positive samples at the Maitland cemetery compared to the other two cemeteries. Furthermore, the higher concentration of human habitats around the Maitland cemetery, compared to Delft and the Welmoed cemeteries, could have also served as non-point sources of pollution in the Maitland cemetery.

There have been controversies on the potential of microbial contamination of groundwater sources originating from cemeteries. Although some studies have reported high levels of bacteria in the wells around cemeteries and have attributed this to burial grounds, others have indicated that there were minimal chances that such contamination originated from the cemetery [33]. In the current study, only two boreholes (M6 and M8), which were found in the Maitland cemetery, recorded mean *E. coli* counts of 11.5 and 5.2 MPN/100 mL, respectively (Table 1). In South Africa, dead bodies are first embalmed using different chemical practices, placed in coffins and then buried directly into the ground at depths of 1.70–2.50 m, which is similar to the situation in many other countries [33,34]. The embalming process typically reduces the microbial content of a dead body and prevents the decomposition of the dead body before the burial [35]. Therefore, this could explain the low number of positive borehole samples and the low mean bacterial count in the positive ones compared to surface water samples. Furthermore, although the decomposition rate of buried cadavers may vary depending on numerous factors, including the presence of a coffin, prevailing temperatures or the presence of clothing on the cadaver, a study in South Africa indicated that cadavers could attain a well-advanced decomposition rate after ±90–183 days following interment [36]. Furthermore, it has been shown that although the microbial population in decomposing bodies may increase over time, there is a decrease in diversity, especially in the gut microbiome, with Clostridia becoming more common due to their ability to withstand the harsh conditions accompanying decomposing cadavers [37]. This could have also accounted for the negative results obtained for the borehole samples as these were collected in areas where the graves were more than three years old. Based on these results, it is logical to assume that the observed bacterial count in the borehole samples was the result of seepage from the polluted surface water bodies. This is further supported by the fact that the cemeteries are located in areas with very shallow water tables, while the constant flooding of the cemetery by sea water could influence the transport of surface pollutants into the subsurface waters.

### 3.2. Virulence Potentials of Isolates

Some pathogenic strains of *E. coli*, such as Enteroaggregative *E. coli* (EAEC), have been classified as emerging bacterial pathogens [38,39]. The different pathogenic *E. coli* strains have been isolated from different aquatic environments, such as rivers [29], lakes [40], seas [41] and even in groundwater resources [42]. In the current study, 48% of the pure isolates carried at least one of the virulence genes tested. For example, considering the Maitland cemetery, which had a large human population living in the surroundings, the presence of these pathogenic forms could pose a potential health threat as the cemetery serves as a reservoir of these bacteria originating from surrounding informal settlements and subsequently, becomes a source of pollution for groundwater resources in the surrounding areas. It is important to note that this Maitland cemetery is characterised by a high water table. Preliminary visits to the cemetery sites revealed that the water tables were at a depth of 1.66–4 m below ground level. Such shallow aquifers together with the intrusion of seawater have been reported to facilitate the contamination of groundwater resources with microbial pathogens [43]. This could account for the low number of isolates with pathogenic potentials observed in the borehole samples compared to the surface water samples in the current study. Although most of the isolates (52%) were negative for the genes tested and that none of the isolates was identified as belonging to the ETEC group, it should be noted that this could have been due to the selected genes tested. Virulence in members of the ETEC group is characterised by the presence of either the *ST* gene (coding for the production of a heat-stable toxin), the *LT* gene (coding for the production of a heat-labile toxin) or a combination of both genes [44]. In the present study, only the *ST* gene was tested. This could have also reduced the chances of detecting other ETEC members that could be *ST*-negative but *LT*-positive.

### 3.3. Antibiotic Resistance Profiles of Isolates

The fight against antibiotic resistance is far from being resolved as bacteria are increasingly becoming resistant even to newly developed formulations. In the current study, 100 *E. coli* isolates were tested for resistance to eight antibiotics belonging to eight different classes. The majority (87%) of these isolates were resistant to at least one of the antibiotics tested. More isolates were resistant to Trimethoprim and the aminoglycoside Streptomycin. Resistance to Streptomycin has been reported in food animals [45] and hospital settings [46] for many years. For example, in birds, it has been reported that some strains of avian pathogenic *E. coli* (APEC) possessed a plasmid with genes that encoded resistance to numerous antibiotics, including Streptomycin [45]. It has also been reported that due to the location of this gene in a plasmid, its transfer to other *E. coli* strains and related organisms is very likely [47]. Similarly, it has recently been demonstrated that the genes present in class 1 and class 2 integrons in APEC encode for resistance to Streptomycin (through adenyltransferase) and Trimethoprim (through a dihydrofolate reductase) and that these genes have been detected in both pathogenic and non-pathogenic *E. coli* from animal, human and environmental samples [48]. Therefore, this could explain the highest resistance of the isolates in this study to the two drugs.

Ceftolozane/Tazobactam (C/T) is a recently developed cephalosporin-β-lactamase inhibitor that has been proven to be effective against a broad range of organisms. Ceftolozane in C/T is designed to be effective against multi-drug resistant *Pseudomonas aeruginosa* and many *Enterobacteriaceae* although its activity is compromised by Extended-Spectrum Beta-Lactamases (ESBL)-producing organisms [49]. However, the coupling of Ceftolozane with Tazobactam (a β-lactamase inhibitor) has facilitated the treatment of many infections, including those caused by ESBL-producing bacteria [50]. Despite the enhanced potency of this drug combination, the resistance of some clinical isolates, including *E. coli*, has been reported [51,52]. In our study, we observed that a surprisingly high percentage (52%) of our isolates were resistant to the drug formulation. To the best of our knowledge, this is the first report on resistance to C/T recorded in environmental samples. Although this could not easily be explained, it is suggested that in addition to exposure to antibiotics in the environment, other stressors, such as the exposure to heavy metals, could contribute to an increased antibiotic resistance in environmental strains [53]. This could further justify the fact that 86.75% of the resistant isolates were multi-drug resistant, exhibiting resistance to three or more of the antibiotics tested. Heavy metals have been reported to co-select for antibiotic resistance in the environment [54]. Furthermore, cemeteries have been reported to be reservoirs of high levels of heavy metals, which probably originates from the degraded coffin materials [24,55]. Based on this, we would have expected to record higher levels of resistance in the borehole isolates compared to the surface water samples. However, this was not the case in the current study as only eight of the 87 resistant isolates were from the boreholes. Furthermore, the antibiotic resistance profiles displayed by these eight isolates were more prevalent in the surface sample isolates. Therefore, it is probable that the resistant isolates obtained from the borehole samples could have filtered down from the polluted surface water into the shallow groundwater.

The isolation of antibiotic-resistant *E. coli* from the cemetery environment in the current study indicates that cemeteries are potential reservoirs of multi-drug resistant pathogenic bacteria. These organisms most likely originated from the surrounding informal settlements and could represent a potential health risk for grave diggers. In places with shallow aquifers, such as in the study area, these organisms could eventually contaminate groundwater resources through soil infiltration, thus posing a significant health threat to the surrounding populations. However, given that surface water samples were more polluted with these potentially pathogenic and antibiotic-resistant *E. coli* isolates than borehole water samples, further studies are needed to understand whether burial practices could contribute to their presence and survival in the cemetery environment, and how long they can survive in such environments. Although further studies need to be conducted to better understand the dynamics of microbial pathogens in a cemetery environment and the potential role of cemeteries in determining the microbial quality of groundwater, we recommend that the selection of areas for use as cemeteries should involve full hydrological assessment to prevent any risk of contamination of surrounding water resources.

## 4. Materials and Methods

### 4.1. Study Area

The present study was conducted in Cape Town, which is located in the Western Cape province of South Africa. For this study, three cemeteries were selected, which were namely the Delft, Maitland and Welmoed Cemeteries. These span approximately 3.7 ha, 114.2 ha and 94 ha, respectively (Figure 1). All three cemeteries are located in an area known as the Cape Flats. The Cape Flats covers a surface area of about 600 km^2^ and is comprised of fluvial, marine and aeolian Tertiary and Quaternary sedimentary deposits, which are up to 50 m thick in some places [56]. The Cape Flats Aquifer is a primary intergranular aquifer, which is almost wholly saturated as groundwater levels are within a few meters of the ground level. In certain cases, the water table intersects the ground surface to form wetlands or marsh areas [57].

### 4.2. Sample Collection

**Figure 2:**
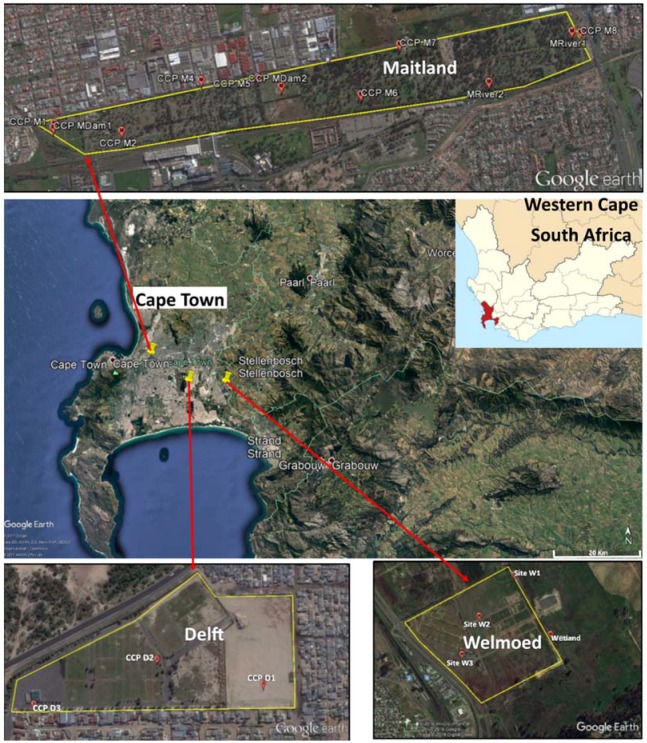
Location of the three cemeteries with the sample points (Google Earth).

Samples were collected from three cemeteries in Cape Town, which were namely Welmoed, Delft and Maitland (Figure 2). The number of sampling points was determined by the size of the cemetery and the ease of accessibility. However, an attempt was made to cover both areas of recent burial and those of old burials. For each sampling point, a hand auger was used to bore holes until the water table was intersected and the depth of each borehole was measured and recorded. After this, each borehole was allowed to stand for a few minutes for the water to collect and stabilise. Once stabilised, water was extracted from each hole using a brailer. A new brailer was used for each borehole. Surface water samples were also collected from some “wetlands” and “streams” identified in each cemetery. These wetlands were patches of water collected in depressions in the cemetery and surrounded by vegetation. The streams were small water bodies flowing through the cemetery. Water samples were collected following the protocol described in the Groundwater Sampling Manual [58]. The 500-mL water samples were collected in triplicate per sampling site for microbiological analysis. Samples were transported on ice to the microbiology laboratory of the Council for Scientific and Industrial Research (CSIR), Stellenbosch, and analysed within 6 h from the time of collection.

### 4.3. Enumeration of Escherichia coli

Isolation and identification of *E. coli* were carried out using the Colilert^®^ 18/Quanti-Tray^®^/2000 systems (IDEXX Laboratories, Inc., Johannesburg, South Africa) following the manufacturer’s instructions. Upon incubation of the sealed Quanti-Trays at 37 °C for 18–24 h as recommended by the manufacturer, pure isolates were obtained as previously described [59]. Briefly, the back of a positive Quanti-Tray was cleaned using 70% ethanol and then punctured using a sterile disposable inoculation loop. After this, a loopful of the tray’s content was inoculated onto Eosin Methylene Blue (EMB) agar (Merck, Modderfontein, South Africa) and incubated at 37 °C for 24 h. Five colonies were randomly selected from each plate. For plates with ≤5 colonies, all the isolates were selected for further purification. Following incubation, a single colony was selected and streaked further on EMB agar to obtain pure isolates, which were then used for virulence and antibiotic susceptibility profiling.

### 4.4. Determination of Virulence Potentials of Isolates

Single colonies of the isolates were inoculated in nutrient broth and incubated at 37 °C overnight in a shaking incubator. For the determination of the virulence properties of the isolates, 1 mL of the nutrient broth culture was harvested by centrifugation at 13,000 rpm and DNA was extracted from the pellet as previously described [60]. The extracted DNA was used as the template DNA in different real-time PCR assays for the identification of genes associated with virulence in 6 *E. coli* pathotypes. The various genes tested and the associated pathotypes are shown in Table 4.

Before the determination of the virulence potentials, each isolate was first confirmed as *E. coli* by testing for the housekeeping gene (the malate dehydrogenase gene), which is present in all *E. coli* strains [61]. The primers and PCR conditions used for the various genes were previously described by Abia et al. [62]. Positive controls used in the PCR assays included EPEC B170, EIEC ATCC 43892, EAEC 3591-87, EHEC O157:H7, ETEC DSM 10973 and NMEC DSM 10819. All controls were obtained from the CSIR Microbiology Laboratory in Pretoria. Reaction mixtures without DNA, which were used as No-template controls, were also included in each PCR assay.

All PCR assays were performed on a Corbett Life Science Rotor-Gene™ 6000 Cycler (Qiagen, Hilden, Germany). Melt curve analyses for the real-time determination of the presence or absence of the genes of interest were performed using the Rotor-Gene™ real-time analysis software (version 6.1, build 93; Corbett Life Science (Pty) Ltd., Sydney, Australia).

### 4.5. Determination of Antibiotic Resistance Profiles

Following incubation in nutrient broth, the turbidity of the broth culture was adjusted to a 0.5 McFarland standard before inoculating onto Muller Hinton Agar. After this, we tested for resistance to eight antibiotics (belonging to eight different antibiotic classes) using the disk diffusion method as previously described [63]. The antibiotics tested included a phenicol (Chloramphenicol—C 25 µg), Tetracycline (T 25 µg), cephalosporin (Cephalexin—CFX 30 µg), folate pathway inhibitor (Trimethoprim—TM 5 µg), fluoroquinolone (Ciprofloxacin—CIP 5 µg), nitrofuran (Nitrofurantoin—NI 300 µg), cephalosporin-β-lactamase inhibitor (Ceftolozane/Tazobactam—C/T 40 µg) and an aminoglycoside (Streptomycin—S 10 µg). Zones of inhibition around each disc were measured after 24 h incubation at 37 °C and compared to reference values as recommended by the Clinical and Laboratory Standards Institute [64].

## 5. Conclusions

The current study suggests that cemeteries are potential environmental reservoirs of multi-drug resistant pathogenic bacteria. While further studies are needed to understand the dynamics of these drug-resistant pathogenic organisms in such environments, and whether burial practices could favour their extended survival in cemeteries, selection of sites for the burial of human remains should take into consideration the drainage network of the area to avoid potential contamination of nearby water sources. Such pollution, should it happen, could have far-reaching public health consequences. 

## Figures and Tables

**Table 1:** Mean *Escherichia coli* count for water samples collected at three Cape Town cemeteries.

Cemetery	Sample Site *	Depth (m) **	Replicate Samples (MPN/100 mL)			Geometric Mean (MPN/100 mL)	Standard Deviation
			1	2	3		
Delft	D2	3.1	<1	<1	<1	0.0	0.0
	D3	na	<1	<1	<1	0.0	0.0
Maitland	MDam1	na	1	1	4.1	1.6	1.8
	MDam2	na	1	4.1	1	1.6	1.8
	MRiver1	na	2419.6	2419.6	2419.6	2419.6	0.0
	MRiver2	na	2419.6	2419.6	2419.6	2419.6	0.0
	M1	1.6	<1	<1	<1	0.0	0.0
	M2	2.8	<1	<1	<1	0.0	0.0
	M4	4	<1	<1	<1	0.0	0.0
	M5	2.5	<1	<1	<1	0.0	0.0
	M6	2	9.7	18.7	8.4	11.5	5.6
	M7	2.7	<1	<1	<1	0.0	0.0
	M8	2.3	8.6	3.1	5.2	5.2	2.8
Welmoed	Wetland	na	65.7	86.5	42	62.0	22.3
	W2	1.8	<1	<1	<1	0.0	0.0
	W3	2.4	<1	<1	<1	0.0	0.0

* D: Delft; M: Maitland; W: Welmoed. ** na: not applicable (samples were collected from the surface water bodies, i.e., streams and wetlands).

**Table 2:** Distribution of isolates with respect to resistance to each antibiotic tested *.

Profile	Number of Isolates							
	C (25 µg)	S (10 µg)	T (25 µg)	CFX (30 µg)	TM (5 µg)	CIP (5 µg)	NI (300 µg)	C/T (40 µg)
Susceptible	71	26	38	65	29	53	66	48
Intermediate	3	3	0	0	0	2	6	0
Resistant	26	71	62	35	71	45	28	52

* C: Chloramphenicol; S: Streptomycin. T: Tetracycline; CFX: Cephalexin; TM: Trimethoprim; CIP: Ciprofloxacin; NI: Nitrofurantoin; C/T: Ceftolozane/Tazobactam.

**Table 3:** Phenotypic profiles of antibiotic-resistant isolates.

Number of Antibiotic Resistance	Antibiotic Resistance Profile	Number of Isolates
1	C/T	1
2	S-C/T	3
	S-T	5
	TM-C/T	1
	TM-CIP	1
3	S-CFX-C/T	1
	S-T-C/T	2
	S-T-CFX	1
	S-TM-C/T	4
	T-TM-C/T	1
	T-TM-CIP	3
4	C-CFX-TM-NI	2
	C-S-T-C/T	2
	S-CFX-TM-C/T	1
	S-TM-CIP-C/T	1
	S-TM-CIP-NI	1
	S-T-TM-C/T	1
	S-T-TM-CIP	13
	T-CFX-TM-CIP	1
5	C-CFX-TM-CIP-NI	1
	C-S-CFX-NI-C/T	1
	C-S-T-TM-C/T	1
	C-T-CFX-CIP-C/T	1
	S-CFX-TM-NI-C/T	1
	S-T-CFX-TM-CIP	2
	S-T-CFX-TM-C/T	2
	S-T-TM-CIP-C/T	8
6	C-S-CFX-TM-CIP-C/T	1
	C-S-CFX-TM-NI-C/T	1
	C-S-T-CFX-TM-C/T	1
	C-S-T-TM-NI-C/T	1
	C-T-CFX-TM-NI-C/T	1
	S-T-CFX-TM-NI-C/T	2
	S-T-TM-CIP-NI-C/T	1
	T-CFX-TM-CIP-NI-C/T	1
7	C-S-CFX-TM-CIP-NI-C/T	1
	C-S-T-CFX-TM-CIP-C/T	2
	C-S-T-CFX-TM-CIP-NI	4
	C-S-T-CFX-TM-NI-C/T	4
	C-S-T-TM-CIP-NI-C/T	1
8	C-S-T-CFX-TM-CIP-NI-C/T	4

**Table 4:** Virulence genes investigated and associated *Escherichia coli* pathotypes.

Designation	*E. coli* Pathotype	Genes Targeted
EPEC	Enteropathogenic *E. coli*	*eaeA*
EHEC	Enterohaemorrhagic *E. coli* (or STEC)	*eaeA*, *stx1*, *stx2*
EAEC	Enteroaggregative *E. coli*	*eagg*
EIEC	Enteroinvasive *E. coli*	*ipaH*
ETEC	Enterotoxigenic *E. coli*	*ST*
NMEC	Neonatal Meningitis *E. coli*	*ibeA*
